# In vivo evaluation of CD38 and CD138 as targets for nanoparticle-based drug delivery in multiple myeloma

**DOI:** 10.1186/s13045-020-00965-4

**Published:** 2020-11-02

**Authors:** David T. Omstead, Franklin Mejia, Jenna Sjoerdsma, Baksun Kim, Jaeho Shin, Sabrina Khan, Junmin Wu, Tanyel Kiziltepe, Laurie E. Littlepage, Basar Bilgicer

**Affiliations:** 1 Department of Chemical and Biomolecular Engineering, University of Notre Dame, 205C McCourtney Hall, Notre Dame, IN 46556-5637 USA; 2 Department of Chemistry and Biochemistry, University of Notre Dame, Notre Dame, IN 46556 USA; 3 Harper Center Research Institute, University of Notre Dame, Notre Dame, IN 46556 USA; 4 Advanced Diagnostics and Therapeutics, University of Notre Dame, Notre Dame, IN 46556 USA

**Keywords:** Liposomes, Peptide-targeted, Multiple myeloma, Drug-loaded, Nanoparticle, CD38, CD138, Biodistribution, Efficacy

## Abstract

**Background:**

Drug-loaded nanoparticles have established their benefits in the fight against multiple myeloma; however, ligand-targeted nanomedicine has yet to successfully translate to the clinic due to insufficient efficacies reported in preclinical studies.

**Methods:**

In this study, liposomal nanoparticles targeting multiple myeloma via CD38 or CD138 receptors are prepared from pre-synthesized, purified constituents to ensure increased consistency over standard synthetic methods. These nanoparticles are then tested both in vitro for uptake to cancer cells and in vivo for accumulation at the tumor site and uptake to tumor cells. Finally, drug-loaded nanoparticles are tested for long-term efficacy in a month-long in vivo study by tracking tumor size and mouse health.

**Results:**

The targeted nanoparticles are first optimized in vitro and show increased uptake and cytotoxicity over nontargeted nanoparticles, with CD138-targeting showing superior enhancement over CD38-targeted nanoparticles. However, biodistribution and tumor suppression studies established CD38-targeted nanoparticles to have significantly increased in vivo tumor accumulation, tumor cell uptake, and tumor suppression over both nontargeted and CD138-targeted nanoparticles due to the latter’s poor selectivity.

**Conclusion:**

These results both highlight a promising cancer treatment option in CD38-targeted nanoparticles and emphasize that targeting success in vitro does not necessarily translate to success in vivo.

## Background

Multiple myeloma is a plasma cell malignancy that develops solid tumors within the protective microenvironment of the bone marrow [1, 2]. These malignant B cells form bone lesions and produce misfolded paraproteins that result in frequent infections, anemia, amyloidosis, and kidney failure [3, 4]. Multiple myeloma globally affects approximately 500,000 people and causes roughly 100,000 deaths annually [5]. While currently treatable, multiple myeloma remains incurable and has a five-year survival rate of 49% [6]. Current treatment of multiple myeloma typically involves high-dose chemotherapy, which is often associated with improved outcomes but often comes with major side effects that can limit dosages [7]. As a common occurrence, multiple myeloma relapses after treatment, becoming resistant to previously effective treatments [8]. This relapsed multiple myeloma typically is treated with other chemotherapy, such as proteasome inhibitors or imide drugs, and recently approved monoclonal antibodies, such as elotuzumab or daratumumab [9–13]. The bone marrow microenvironment causes the recurrent cancer cells to develop cell adhesion-mediated drug resistance (CAM-DR), but recent animal studies have shown that this resistance can be overcome through the use of actively targeted therapies [2, 14]. Thus, there has been significant recent effort toward the use of ligand-targeted nanoparticles for drug delivery for multiple myeloma with the main objective of overcoming drug resistance and improving patient outcome [14–16].

Our laboratory has previously researched the use of targeted nanoparticles for treatment for multiple myeloma, particularly in the field of peptide-targeted liposomes. We have previously identified overexpressed receptors common to multiple myeloma cells, such as VLA-4 and LPAM-1, and developed and optimized targeted formulations with which to deliver a drug payload [17–19]. These systems showed significantly increased uptake over that of nontargeted particles in vitro and in vivo. Nevertheless, not every multiple myeloma case has overexpressed VLA-4 or LPAM-1 receptors. Therefore, developing liposomal nanoparticle formulations that target other receptors of significance in this disease will increase the available therapies available to fight this disease and to improve patient outcomes [20–22].

Two receptors, CD38 and CD138, have recently garnered much interest for multiple myeloma. CD38 is the target of the recently FDA-approved monoclonal antibody daratumumab, which makes it an ideal receptor to evaluate for targeted drug delivery therapies [9, 11, 23, 24]. CD138, also known as Syndecan-1, has also been recently investigated as a potential target for antibody-conjugated drug therapies for multiple myeloma as well as in a wide variety of cancer types, including bladder cancer and triple-negative breast cancer due to overexpression in these malignancies [25–28]. While peptide-targeted nanoparticles have never been used before for either of these promising targets, the unique benefits of a multivalent liposomal targeting system could be effective in treating multiple myeloma.

Peptide-targeted liposomes hold a variety of benefits that make them an ideal nanoparticle system for selective cancer drug delivery [29]. Liposomes are the longest studied and most frequently developed, highly modifiable nanoparticle platform [30–32]. Liposomal delivery systems take advantage of the enhanced permeability and retention (EPR) effect to passively accumulate preferentially at a tumor site [33, 34]. Their lipid bilayer feature also accommodates loading of a variety of drugs, either within the aqueous core, within the hydrophobic lipid bilayer, or in the form of a lipid-conjugated prodrug that is incorporated into the bilayer [35]. Liposomes are typically modified with a polyethylene glycol (PEG) coating to become stealth to an immune response and to reduce clearance by the reticuloendothelial system [36]. They can also be functionalized for active targeting with addition of various molecules, such as antibodies, antibody fragments, small molecules, or peptides [37].

Our group developed a now well-established method of targeted-liposome synthesis to offer uniquely high control over the valency of peptide targeting and increased reproducibility in production between batches [18, 19, 38]. This is accomplished by synthesizing and purifying peptide–lipid conjugates individually prior to particle synthesis and then using the pure building blocks at precise stoichiometric ratios while incorporating into the lipid film before hydration/extrusion, which allows precise control over the valency of the peptide per liposome. We also take advantage of the combined effects of weak to moderate monovalent affinity of a targeting peptide and the simultaneous presentation of multiple copies of it per liposome to provide a multivalent binding effect that we tune to selectively achieve increased avidity for the target of cancer cells over healthy cells, consequently reducing off-target effects.

Previously, we investigated the efficacy of VLA-4-targeted liposomal nanoparticles and found that they can significantly enhance tumor cell uptake in vivo [19]. Furthermore, we also described our strategy for optimizing a nanoparticle formulation designed for selective targeting [16]. Using a similar approach for optimization, in this study, we optimized the uptake and efficacy of two doxorubicin-prodrug-loaded nanoparticle formulations that target two separate receptors using in vitro and in vivo studies. Both biodistribution and efficacy were analyzed in vivo to determine the delivery profile of the nanoparticles as well as their anti-tumor effectiveness. CD38-targeted nanoparticles showed significantly greater tumor cell uptake and enhanced efficacy over CD138-targeted nanoparticles in vivo that would not have been expected from solely the results of the in vitro study. By evaluating and comparing several formulations, we successfully identified the most potent CD38-targeted formulation that clearly outperformed both free drug- and the nontargeted drug-loaded nanoparticles, making them desirable candidates for clinical testing in multiple myeloma tumors that express CD38.

## Methods

### Materials

N-Fmoc-amino acids, Rink Amide resin, 2-(1H-Benzotriazole-1-yl)-1,1,3,3-tetramethyluronium hexafluorophosphate (HBTU), and bovine serum albumin (BSA) were purchased from EMB Millipore (Billerica MA). Fmoc-(EG)_n_–OH reagents were purchased from Quanta Biodesign (Powell, OH). Palmitic acid, cholesterol, *N,N*-diisopropylethylamine (DIEA), trifluoroacetic acid (TFA), triisopropylsilane (TIS), acetonitrile (ACN), 2-propanol (IPA), *N,N*-dimethylformamide (DMF), dichloromethane (DCM), and piperidine were purchased from Sigma-Aldrich (St. Louis, MO). Fluorescein 5-isothiocyanate (FITC) was purchased from Toronto Research Chemicals (Toronto, Canada). FITC antihuman CD38 (HIT2) and FITC antihuman CD138 (MI15) antibodies were purchased from Biolegend (San Diego, CA). 1,2-Distearoyl-*sn*-glycero-3-phosphocholine (DSPC), methoxy PEG2000-DSPE (PEG2000-DPSE), and fluorescein PE were purchased from Avanti Polar Lipids, Inc (Alabaster, AL). 1,1′-Dioctadecyl-3,3,3′,3′-tetramethylindodicarbocyanine perchlorate (DiD), HyClone fetal bovine serum (FBS), and phosphate-buffered saline (powdered, 7.4 pH) (PBS) were purchased from Thermo Fisher Scientific (Waltham, MA). Doxorubicin hydrochloride (Dox) was purchased from Sigma-Aldrich (St. Louis, MO). CCK-8 was purchased from Dojindo Laboratories (Kumamoto, Japan).

### Synthesis of peptides and peptide(K_3_)–EG_linker_–lipid conjugates

Peptides and conjugates were synthesized using solid support Fmoc chemistry on a Rink Amide resin. Residues were activated using HBTU and DIEA in DMF for 3 min prior to addition to the resin, and coupling was monitored using the Kaiser test. Fmoc-protected residues were de-protected with 20% piperidine in DMF three times for 3 min each. After complete synthesis, molecules were cleaved from the resin with 95/2.5/2.5 TFA/H_2_O/TIS twice for 1 h each time. Molecules were then purified using RP-HPLC on an Agilent (Santa Clara, CA) 1200 series system with either a semi-preparative Zorbax C18 column or a Zorbax C3 column with either an ACN gradient or IPA gradient in the mobile phase, respectively. The column was monitored with a diode array detector from 200 to 400 nm wavelengths. Purified product was characterized using a Bruker Autoflex III Smartbeam Matrix-Assisted Laser Desorption Ionization Time of Flight Mass Spectrometer (MALDI-TOF-MS, Billerica, MA).

### Cell culture

H929, MM1S, RPMI, and IM9 were obtained from American Type Culture Collection (Rockville, MD) and were cultured in RPMI 1640 media (Cellgro, Manassas, VA). All lines were supplemented with 10% FBS, 2 mM l-glutamine (Gibco, Carlsbad, CA), 100 U/mL penicillin, and 100 μg/mL streptomycin (Gibco). H929 cells were also supplemented with 55 µM 2-mercaptoethanol and an additional 10% FBS. Cells were split either two (H929, MM1S) or three (RPMI, IM9) times a week down to 150 k cells/mL.

### Receptor expression analysis and binding assays

Cells were placed in blocking buffer (1.5% BSA in PBS) and placed on ice for 30 min. Cells were then incubated on ice with the binding antibody, peptide, or nanoparticles for 1 h or in the case of the nanoparticles a variety of times, washed twice with PBS, and analyzed on a Guava easyCyte 8HT flow cytometer (Millipore, Burlington, MA). Isotype-matched antibodies and scrambled peptide sequences were used as negative controls.

### Nanoparticle preparation

Liposomal nanoparticles were prepared via dry film hydration as follows. A lipid mixture of chloroform stocks of all components was prepared and dried via rotational evaporation to produce a thin film and was then placed in a desiccator overnight to remove any residual solvent. The lipid films were then hydrated with PBS at 65 °C under rotation for 15 min and then extruded at 65 °C through a 0.05 µm polycarbonate filter. Nanoparticles adhered to the following formula (95-*X*–*Y*):5:5:*X*:*Y* DSPC:PEG2000-DSPE:cholesterol:peptide(K_3_)–EG_linker_)–lipid conjugate:DiD/dox–lipid conjugate where *X* was varied between 0.1 and 1% to control peptide density and *Y* was 0.4% for experiments using the DiD tracer and 2% for experiments using the dox–lipid conjugate.

### Characterization of nanoparticles

Particle sizes were measured using DLS analysis via the 90Plus Nanoparticle Size Analyzer (Brookhaven Instruments Corp., Long Island, NY), using 658 nm light observed at a fixed angle of 90° at 20 °C. Confirmation of the components of the nanoparticle formulations was determined by RP-HPLC on an Agilent (Santa Clara, CA) 1200 series system with a Zorbax C3 column with an IPA gradient in the mobile phase. The column was monitored with a diode array detector from 200 to 400 nm wavelengths. Extruded nanoparticles were compared with equivalent samples of the base components to confirm that the resulting formulations were composed of intended ratios of the individual lipids and conjugates and that the stoichiometries that were used for synthesis of the particles were conserved in the final product.

### In vitro nanoparticle uptake assays

Per well, 100,000 cells were plated in a 24-well dish 6 h prior to each experiment. Nanoparticles were prepared with DiD as a fluorescent maker as described in the section that explains nanoparticle preparation and added to the wells at a 100 µM phospholipid concentration and incubated at 37 °C for 1 to 24 h as described in each experiment. After incubation, cells were washed twice with PBS and then incubated for 10 min with 100 µL of 0.25% Trypsin–EDTA solution (Sigma-Aldrich) to cleave off nanoparticles bound to the surface of the cell but not yet endocytosed. After trypsinization, cells were washed once more with PBS and then analyzed via flow cytometry.

### In vitro cytotoxicity assays

Per well, 20,000 cells were plated in a 96-well dish 6 h prior to each experiment. Nanoparticles were prepared with a dox–lipid prodrug and dosed at the experimental concentrations [39]; 46 h later, CCK-8 was added to the wells, and 2 h later the absorbance from each well was measured at 450 nm.

### In vivo biodistribution and tumor cell uptake studies

NOD–SCID male mice (JAX, Ellsworth, Maine) were irradiated with 150 rad and injected subcutaneously with 5 million H929 cells. When tumors reached a volume of 150 mm^3^, mice were distributed randomly into groups and treated intravenously via retro-orbital injection with DiD-labeled or dox–lipid-labeled nanoparticles. Tumor volume was measured via calipers (volume = 0.5 × length × (width)^2^).

For biodistribution studies, mice were killed 24 h after nanoparticle injection, tumors and major organs were weighed, and ex vivo organ imaging was generated using a Kodak Multispectral FX (Kodak, Rochester, NY) with an excitation of 630 nm and an emission of 700 nm. Total fluorescence was calculated by ImageJ and normalized by tumor or organ mass. Excised tumors were then mechanically fragmented and then further treated with disaggregation solution (0.1% collagenase type IV (Life Technologies) and 0.003% DNase I (Sigma-Aldrich) in PBS) for 45 min at 37 °C with slow agitation. Samples were strained through a 40-µm mesh, washed three times, and analyzed via flow cytometry.

### In vivo cytotoxicity study

NOD–SCID male mice were irradiated with 150 rad and injected subcutaneously with 5 million H929 cells. When tumors reached a volume of 100 mm^3^, mice were distributed into groups and treated intravenously via retro-orbital injection with 3 mg/kg dox or nanoparticles with dox–lipid prodrug. Mice were injected five times total on days 1, 3, 5, 7, and 9 after reaching the starting tumor size. Mouse weight and tumor volume were tracked daily for the duration of the study. Mice with a body weight that dropped significantly from their starting value or in which tumors reached an excessive size were killed per IACUC regulations. All remaining mice were killed at the end of the study on day 28. After being killed, all mice were dissected, and the tumor and major organs were weighed.

### Statement of randomness, selection of groups, and blindness, and statistics

Groups within each experiment were assigned randomly. During the in vivo experiments, the investigators were blinded to group allocation during the experiment and collection of data. Statistical significance was determined using two-tailed *p* values.

## Results

### Identification and evaluation of anti-CD38 and anti-CD138 peptides

First, we identified potential peptide sequences for targeting the receptors CD38 and CD138, both of which are frequently expressed on multiple myeloma cancer cells [40–42]. An anti-CD138 targeting peptide (CD138pep, RKRLQVQLSIRT) was reported previously and used to treat male pattern baldness [43]. Later studies used this peptide sequence for in vitro experiments as a targeting moiety aimed at treating various types of cancers including breast, ovarian, and kidney transfer cancer to success in vitro, but it has not been tested to target multiple myeloma [44–46].

**Fig. 1:**
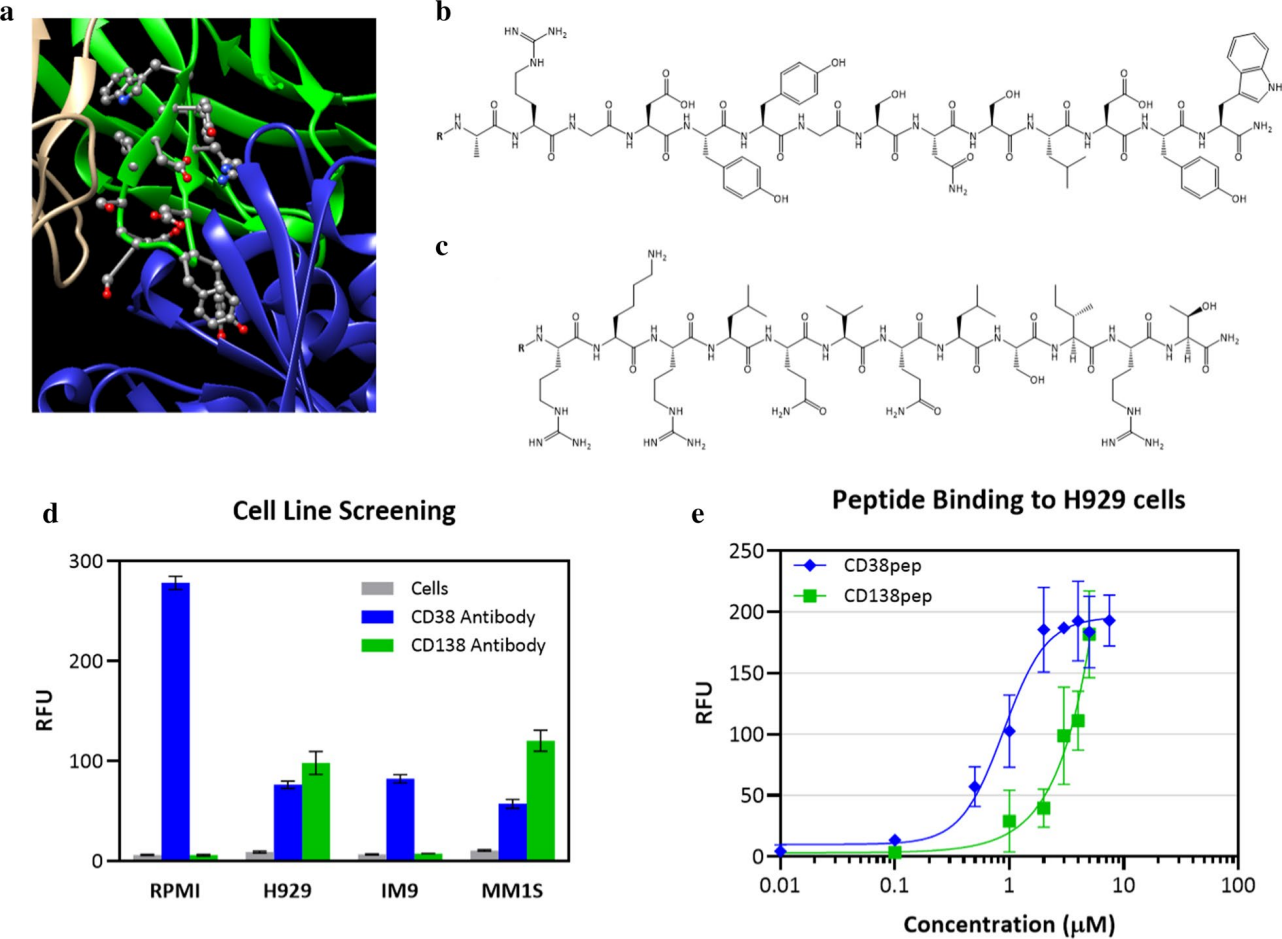
CD38 and CD138 expression in multiple myeloma cell lines and identification of antagonist peptides. **a** Crystal structure of CD38 (blue) binding to the heavy chain of SAR650984-Fab (green), from which CD38pep is derived (PDB# 4CMH). CD38pep replicates the H3 loop of SAR650984-Fab. **b**, **c** Structures of CD38pep (**b**) and CD138pep (**c**), antagonist peptides for CD38 and CD138 respectively. **d** Screening of multiple myeloma cell lines for the presence of CD38 and CD138 receptors using FITC-labeled as determined by flow cytometry. **e** Cellular binding assays using fluorescein-labeled CD38pep and CD138pep. Binding was detected by flow cytometry. Fluorescence of cells incubated without peptides was minimal and subtracted from the presented data. All experiments were done in triplicate. Data represent means (± s.d.)

To identify a peptide that binds to CD38, we studied the crystal structure of an anti-CD38 antibody (Isatuximab, SAR650984-Fab) co-crystallized with CD38 (PDB# 4CMH). We identified a specific peptide sequence on the H3 loop that carried a pronounced role in the binding interaction. We further evaluated this sequence by synthesizing the peptide and carrying out binding experiments to verify its affinity to the CD38 receptor (Fig. 1a). Notably, this paper is the first report of the anti-CD38 peptide (CD38pep, ARGDYYGSNSLDYW).

Both CD38 and CD138 targeting peptides (Fig. 1b, c) were synthesized via solid-support peptide synthesis protocols and purified using RP-HPLC prior to evaluation. Potential cell lines were screened for CD38 and CD138 expression using FITC-labeled anti-CD38 and anti-CD138 antibodies (Fig. 1d). Because the H929 cell line displayed both receptors, it was selected for further evaluation. FITC-labeled versions of CD38pep and CD138pep were synthesized and their binding to H929 cells on ice tested (Fig. 1e). Both peptides showed similar monovalent binding affinities, with CD38pep having a *K*_d_ of 1.0 µM and CD138pep a *K*_d_ of 3.1 µM.

### Design and evaluation of nanoparticles targeted with CD38pep and CD138pep

**Fig. 2:**
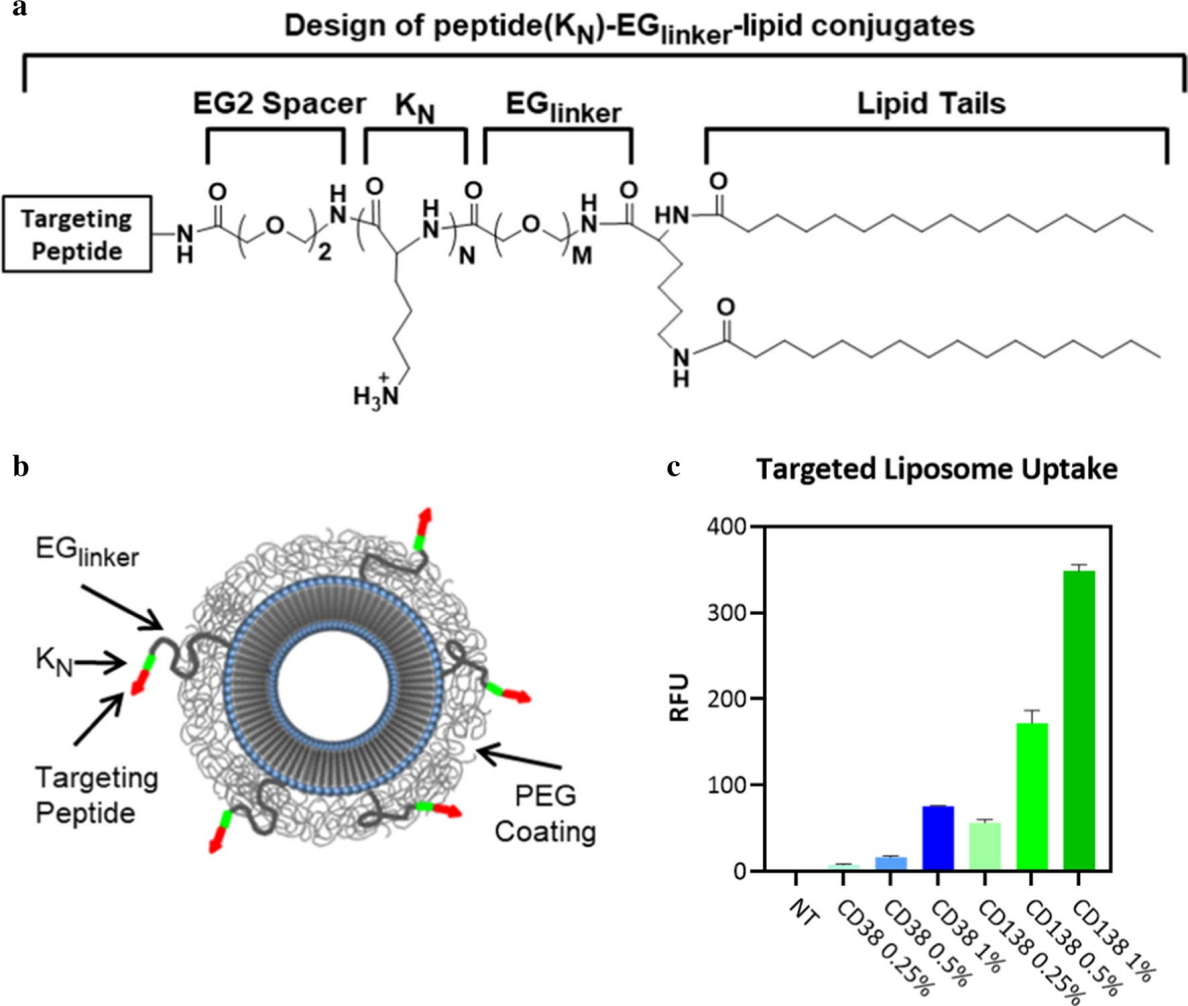
Design of peptide conjugated liposomal nanoparticles and uptake of targeted nanoparticles into H929 myeloma cells. **a** Design of peptide(K_N_)–EG_linker_–lipid conjugates with variable oligolysine (K_N_) content and EG peptide–linker lengths. **b** Schematic of the peptide-targeted nanoparticles. **c** Uptake of nanoparticles targeted with varying densities of CD38pep or CD138pep. Nanoparticles were incubated with H929 cells in media for 3 h, trypsinized to remove nanoparticles bound to the surface but which had not yet undergone cellular uptake, and then fluorescence was measured by flow cytometry. All experiments were done in triplicate. Data represent means (± s.d.)

Peptide–lipid conjugates were developed using a design that our laboratory has thoroughly tested repeatedly with other targeting peptides [15–17]. The targeting peptide sequence was connected to the lipid via an EG2 spacer to a short sequence of three lysines in order to increase the hydrophilicity of the targeting sequence and to promote its exposure above the PEG cloud coating of the nanoparticle surface to improve its availability for receptor binding. This was followed by an EG6 or PEG2000 (approximately 45 ethylene glycol units long) linker to allow the targeting sequence to extend beyond the PEG cloud, and then two lipid tails to allow incorporation into the lipid bilayer of the liposomal nanoparticle (Fig. 2a, b). The peptide–lipid conjugates were synthesized and purified, and then the nanoparticles were prepared using exact stoichiometric ratios of lipid components to ensure consistent targeting peptide density across production batches [16, 47]. Nanoparticles that incorporate the hydrophobic near infrared dye DiD were prepared. Targeted nanoparticle uptake with varying peptide densities was quantified and compared to that of nontargeted nanoparticles using flow cytometry (Fig. 2c). All targeted nanoparticles showed increased uptake over that of nontargeted liposomes with significant increase at the highest peptide densities.

**Fig. 3:**
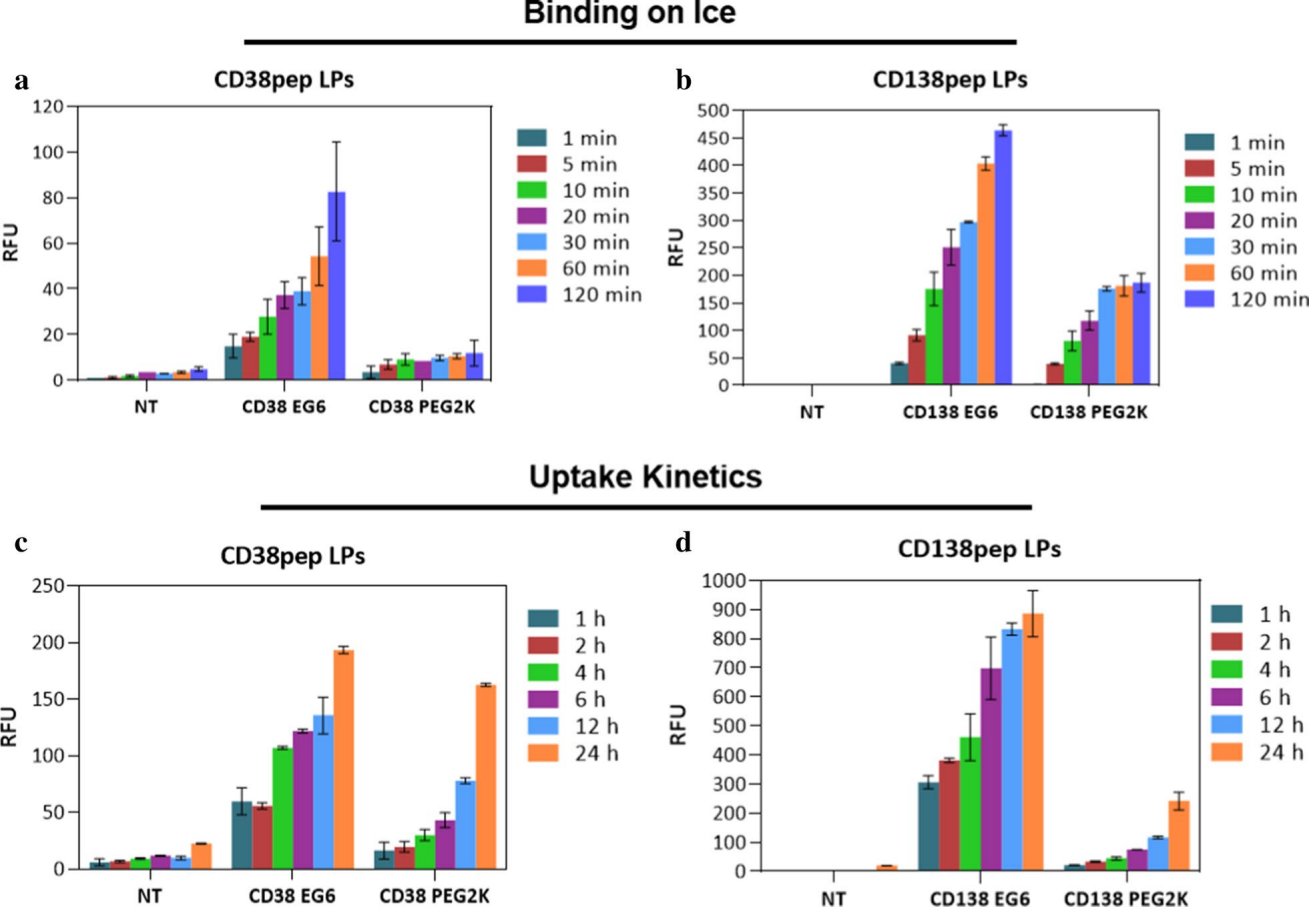
Binding and uptake kinetics of CD38pep- and CD138pep-targeted nanoparticles. **a**, **b** Targeted nanoparticles were incubated with H929 cells on ice for increasing amounts of time. Fluorescence was then measured by flow cytometry. **c**, **d** Targeted nanoparticles with 1% peptide were incubated with H929 cells at 37 °C for increasing amounts of time. The cells were then trypsinized and fluorescence was measured by flow cytometry. All experiments were done in triplicate. Data represent means (± s.d.)

The binding and uptake kinetics of the targeted nanoparticles were then tested with both EG6 and PEG2000 linkers. Our group previously showed in various targeting systems that a longer length linker results in decreased binding and uptake due to greater steric hindering of the binding interaction between targeting peptide and receptor [16, 17, 38]. This observation proved to be generally true for both CD38pep and CD138pep. Cellular binding of the nanoparticles was tested on ice over time (from 1 to 120 min) (Fig. 3a, b). All targeted nanoparticles showed increased binding over nontargeted nanoparticles at each time point. Nanoparticles utilizing the EG6 linker showed higher binding than those using a PEG2000 linker at every time point. The uptake of the nanoparticles at 37 °C was also tested over time (from 1 to 24 h) (Fig. 3c, d). Nanoparticles using an EG6 linker also showed increased uptake over those using the PEG2000 linker for all time points. Subsequent studies used only the EG6 linker formulations due to their increased binding and uptake and more rapid kinetics compared to the PEG2000 formulations. Also, the difference in magnitude between CD38 and CD138 targeted nanoparticles was significant (up to sixfold) for both binding and uptake.

Despite both targeting sequences having similar monovalent affinities, nanoparticles targeted with CD38pep displayed significantly reduced binding and uptake than those targeted with CD138pep. This result could possibly be caused by differences in the receptors that are targeted by each peptide. If CD138 receptors are distributed on the cell surface in a way that encourages multivalent binding, such as arranged in clusters, while CD38 receptors are evenly distributed, then the much higher binding seen when using nanoparticles with CD138pep over those with CD38 would be explained. Additionally, binding to CD138 may trigger more rapid or more prolonged uptake than binding to CD38 due to differences in the cellular pathways that are being activated and lead to an even larger difference in uptake between the two targets.

### Doxorubicin loading and in vitro cytotoxicity

**Fig. 4:**
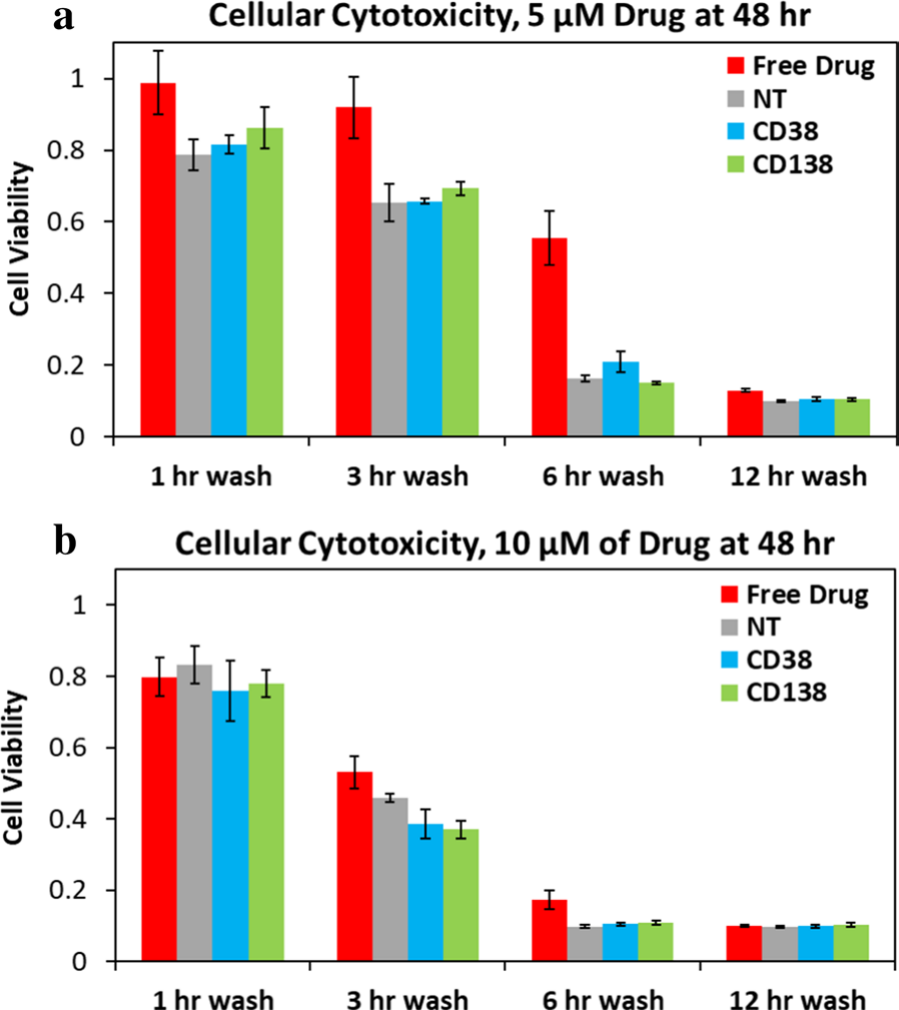
In vitro cytotoxicity of CD38pep- and CD138pep-targeted nanoparticles loaded with a chemotherapeutic. Nanoparticles targeted with 1% peptide density of CD38pep or CD138pep were prepared loaded with doxorubicin prodrug, and their cytotoxicity was tested versus that of nontargeted nanoparticles as well as free doxorubicin. Cells were dosed with either 5 µM (**a**) or 10 µM (**b**) of carfilzomib and allowed to incubate for 1, 3, 6, or 12 h. At this point, the cells were washed to remove all free drug and nanoparticles from the well, fresh media were added, and the cells were incubated for the rest of a 48-h time span at which cell viability was tested with CCK8. All experiments were done in triplicate. Data represent means (± s.d.)

Targeted nanoparticles were then prepared using a pro-drug version of doxorubicin previously studied by our laboratory [39]. By using a doxorubicin pro-drug instead of the standard method of doxorubicin loaded via post-insertion into the core of the liposomal nanoparticle, we were able to ensure that the drug loading of the targeted nanoparticles was consistent between different formulations and batches of nanoparticles, improving the reliability and reproducibility of our studies. This pro-drug includes a pH-sensitive bond between doxorubicin and a lipid tail, allowing for incorporation into the liposomal nanoparticle during extrusion and release once the nanoparticles are endocytosed into the acidic endosome environment within the target cells. The cytotoxicity of the drug-loaded, targeted nanoparticles was then tested against the cytotoxicity of drug-loaded, nontargeted nanoparticles and of the free drug using a pulse cytotoxicity assay (Fig. 4). The cells were incubated with either nanoparticles or drug for 1 to 12 h, at which point cells were washed and placed back in media for 48 total hours, and cell viability was measured. All nanoparticle formulations increased the cellular cytotoxicity over the free drug up until the 12-h wash time point, at which point the free drug was able to passively accumulate within the cells to a high enough level to deliver an equal cytotoxic effect.

### In vivo biodistribution of DiD-labeled targeted nanoparticles

**Fig. 5:**
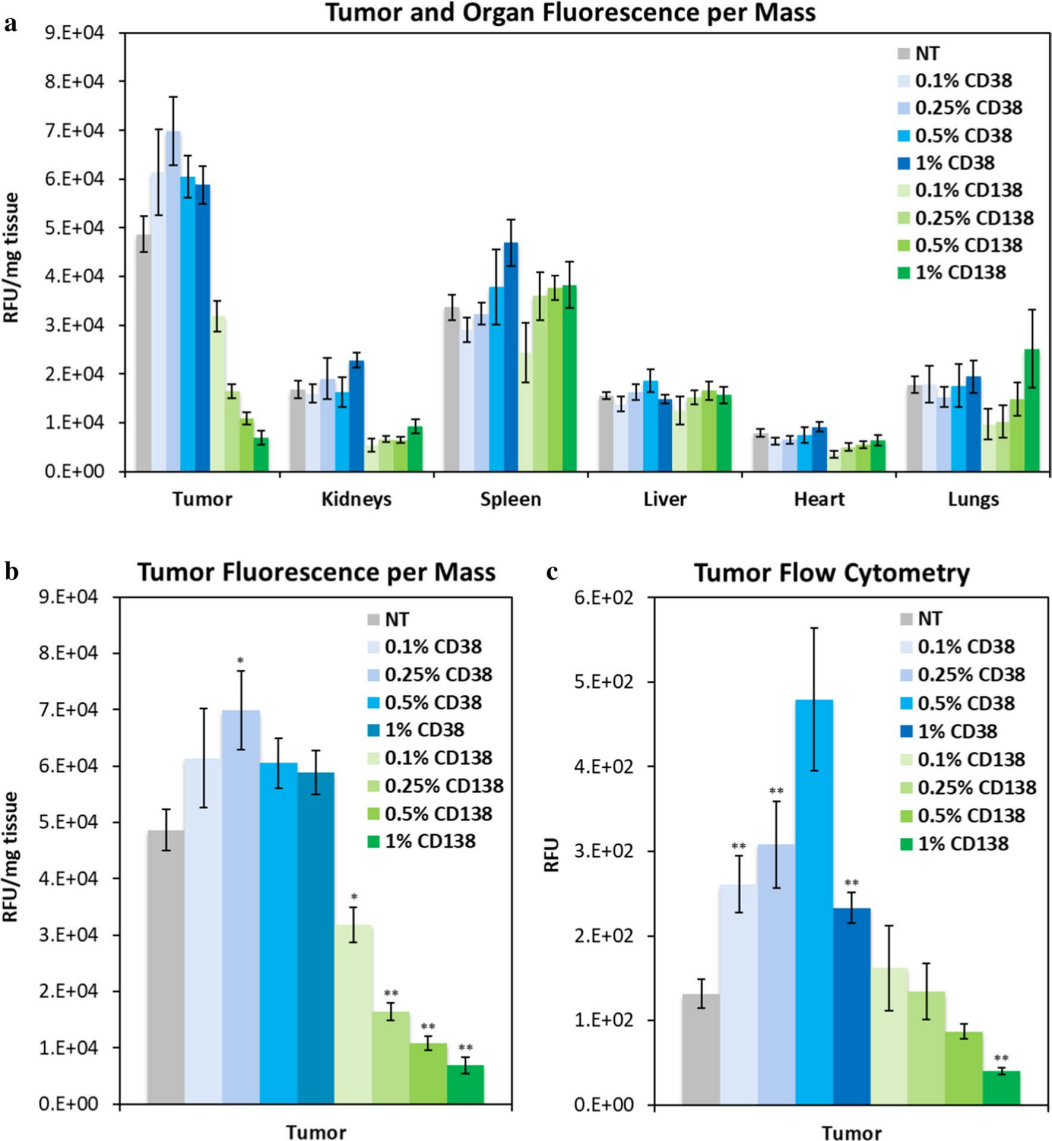
In vivo biodistribution of CD38pep- and CD138pep-targeted nanoparticles loaded with a NIR dye. Nanoparticles targeted with CD38pep or CD138pep were prepared loaded with the NIR dye DiD, and their in vivo biodistribution was tested in a subcutaneous xenograft mouse model of myeloma. Mice were injected with H929 cells and tumors allowed to grow to a predetermined size before IV injection of nanoparticle formulations; 24 h after nanoparticle injection, the mice were killed, and the tumor and major organs were imaged (**a**, **b**). Tumors were digested, and uptake of the nanoparticles by the tumor cells was measured via flow cytometry (**c**). *n* ≥ 5 for all groups. Data represent means (± s.e.). Statistical significance represents comparison to nontargeted liposomes, *p ≤ 0.05, **p ≤ 0.005

To determine the ideal parameters for both CD38- and CD138-targeted nanoparticle formulations, in vivo biodistribution studies of nanoparticles were analyzed using a subcutaneous xenograft mouse model of multiple myeloma. Nanoparticles were loaded with a range of peptide densities from 0.1 to 1%, and all formulations used the EG6 linker. NOD–SCID mice were injected with H929 cells, and tumors grew until they reached approximately 50–100 mm^3^ in volume, at which point the nanoparticles were administered via intravenous retro-orbital injection. After 24 h, the mice were killed and dissected, and the tumors and major organs were imaged to determine nanoparticle accumulation (Fig. 5a, b).

The results of this study first showed that, while treatment with the CD38-targeted nanoparticles showed increased accumulation at the tumor site compared to nontargeted nanoparticles, the CD138-targeted nanoparticles showed significantly decreased tumor accumulation, with even lower accumulation at increased CD138pep densities. As tumor accumulation is mostly due to the passive targeting of the EPR effect, this large decreased accumulation is likely due to off-target binding of the CD138-targeted nanoparticles to other nontumor cells, caused by their particularly high avidity that was observed in vitro. Similar observations were reported with other peptide-targeted nanoparticles, such as VLA-4, where increased peptide density also led to decreased tumor accumulation [16]. Notably, the targeted nanoparticles showed no significant increase in accumulation over the nontargeted nanoparticles across any of the major organs imaged.

After imaging, the tumors were digested, and the nanoparticle uptake by the tumor cells was measured via flow cytometry (Fig. 5c). Tumor cell uptake of CD38-targeted nanoparticles increased over nontargeted nanoparticles, with increasing uptake of nanoparticles formulated with 0.1–0.5% peptide density, with a large drop occurring between 0.5 and 1% peptide density. This drop is likely due to a phenomenon known as the binding-site barrier. When a high-avidity targeted particle encounters a tumor, it is likely to bind strongly to the first cancerous cell it reaches [48, 49]. This leads to the layer of tumor cells surrounding the capillaries absorbing most of the particles, while very few or none makes it to the cells deeper within the tumor. This effect has also been observed with other peptide-targeted nanoparticles [16, 49]. In the case of the CD138-targeted nanoparticles, low tumor cell uptake is likely due to a combination of their low accumulation at the tumor site and the binding-site barrier. Because of these results, we determined whether the CD38-targeted nanoparticles had a noticeable break point in vitro between 0.5 and 1% peptide density (see Additional File 1: Figure S1). The results showed a large increase in uptake between 0.7 and 0.8% peptide density. Based on this information, we formulated 0.7% CD38pep and 0.1% CD138pep for the next part of the study.

### In vivo biodistribution and efficacy of doxorubicin pro-drug-loaded targeted nanoparticles

**Fig. 6:**
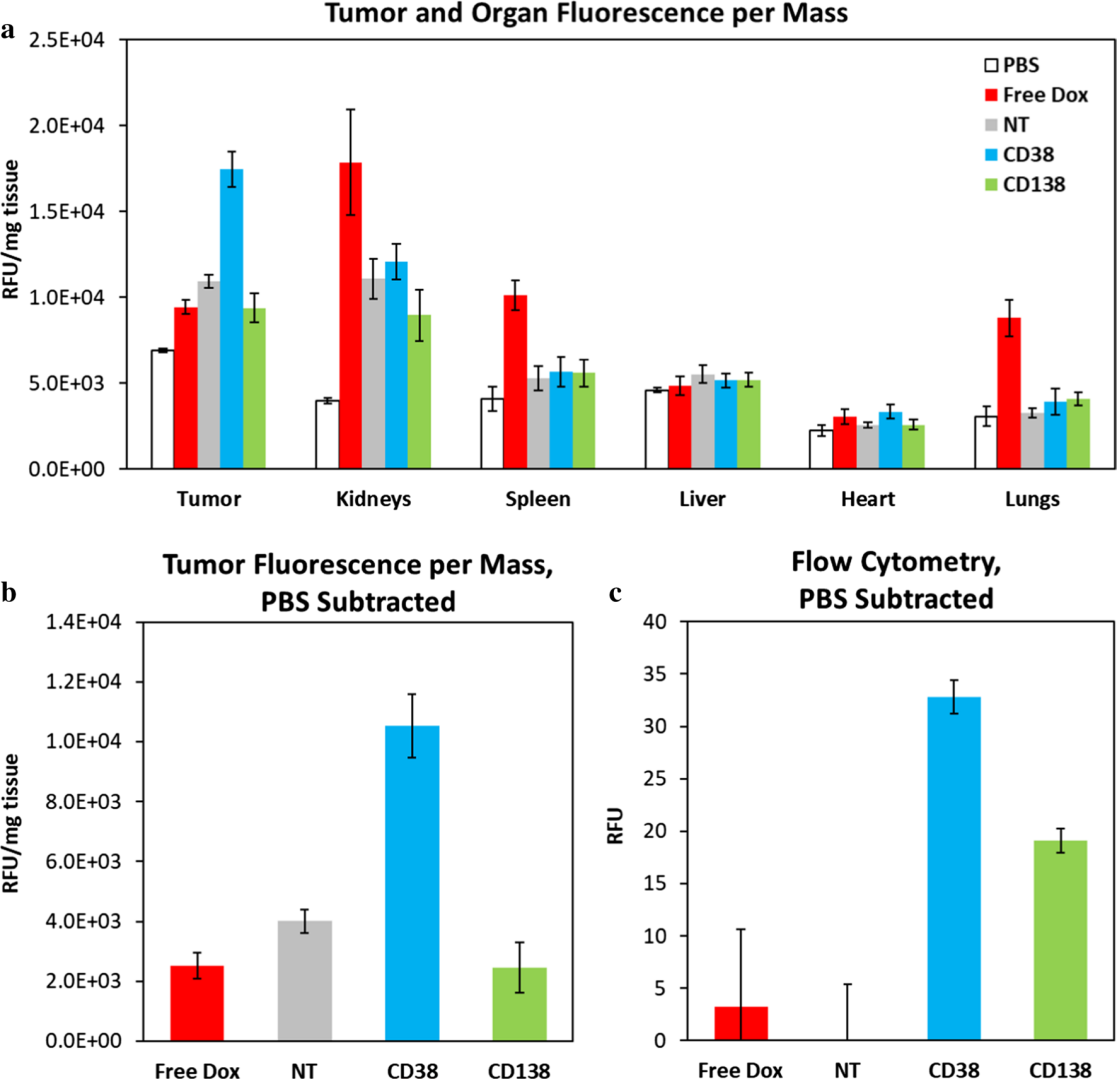
In vivo biodistribution of CD38pep- and CD138pep-targeted nanoparticles loaded with pro-drug doxorubicin. Nanoparticles targeted with CD38pep or CD138pep were prepared loaded with a doxorubicin pro-drug, and their in vivo biodistribution was tested against that of free doxorubicin in a subcutaneous xenograft mouse model. Mice were injected with H929 cells, and tumors grew to a predetermined size before IV injection of nanoparticle formulations. 24 h after nanoparticle injection, the mice were perfused, killed, and the tumor and major organs imaged (**a**, **b**). Tumors were then digested, and uptake of the nanoparticles to tumor cells was measured via flow cytometry (**c**). *n* = 6 for all groups. Data represent means (± s.e.)

The next step was to utilize the optimal formulations of CD38- and CD138-targeted nanoparticles to deliver doxorubicin pro-drug to the tumor. Since doxorubicin is fluorescent, we completed another biodistribution study with the drug-loaded nanoparticles and compared the results with those of free doxorubicin, with the one major difference being that in this study the mice were perfused with PBS. Upon imaging, all nanoparticle groups showed largely decreased drug accumulation in the kidneys, spleen, and lungs over that of the free drug (Fig. 6a). After subtraction of the control group that was injected with PBS, the nontargeted nanoparticles showed approximately 60% higher accumulation at the tumor site than that of the free drug, an increase in a level commonly seen in the literature (Fig. 6b) [33, 34]. Importantly, the CD38-targeted nanoparticles showed 2.6-fold increase in tumor accumulation over that of the nontargeted nanoparticles and a 4.2-fold increase over the free drug. The tumors were enzymatically digested, and uptake was measured via flow cytometry (Fig. 6c). CD38-targeted nanoparticles displayed significantly increased tumor cell uptake over both nontargeted nanoparticles and the free drug. With these results supporting the effectiveness of the CD38pep formulation, we then decided to begin an in vivo efficacy study using these groups.

NOD–SCID mice were injected with H929 subcutaneously, as in the biodistribution studies. The tumors were allowed to grow until a predetermined size (day 0), at which point injections began. Mice were injected with 3 mg/kg of doxorubicin or nanoparticle pro-drug equivalent on days 1, 3, 5, 7, and 9. Tumor volume, survival, and mouse weight were all tracked until tumor volume grew too large or the mouse weight fell too low, at which point the mouse was killed. All surviving mice were killed on day 28. All mice were dissected after being killed, whenever it occurred, and their organs were weighed to determine systemic toxicity.

**Fig. 7:**
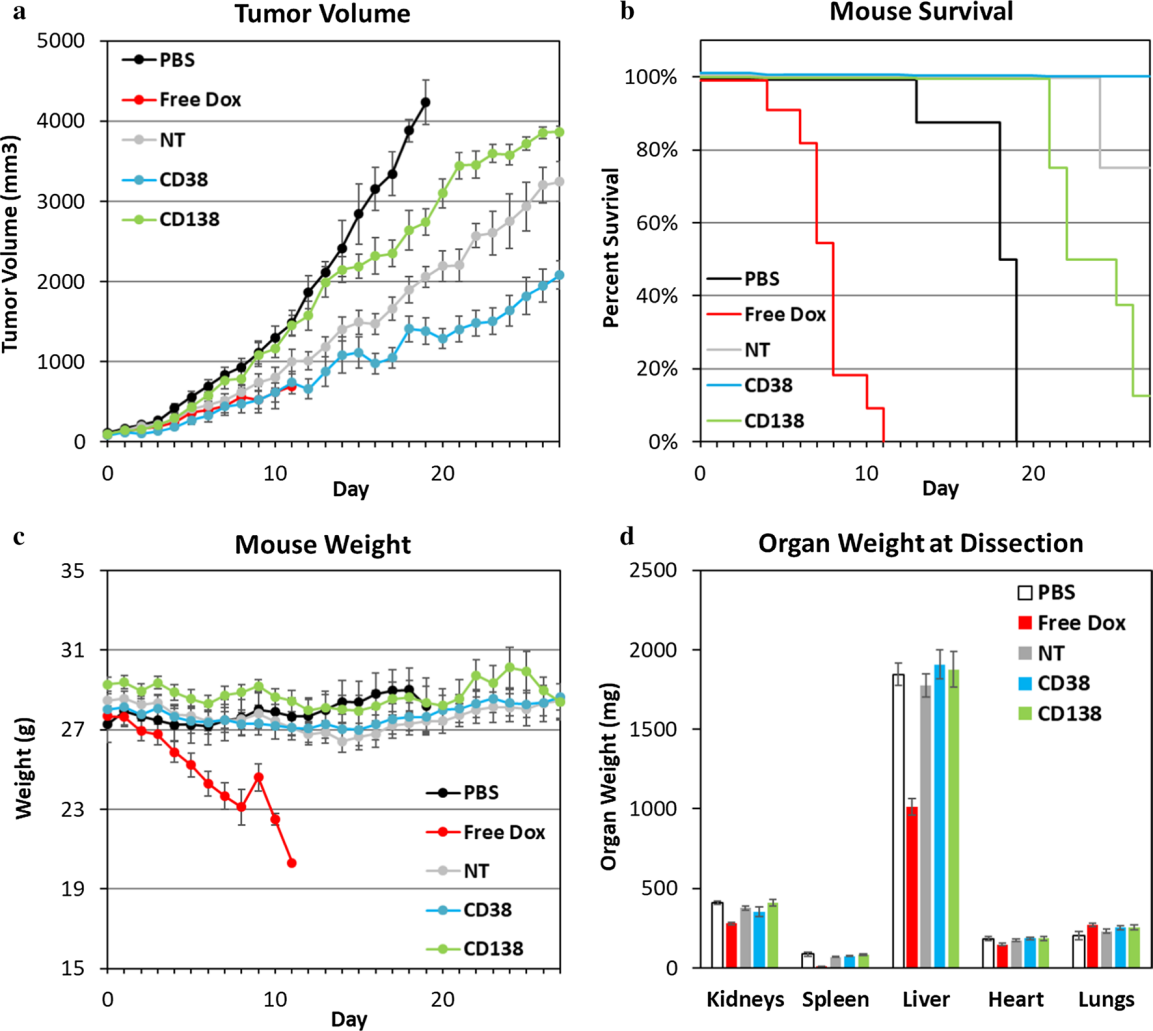
In vivo efficacy of CD38pep- and CD138pep-targeted nanoparticles loaded with pro-drug doxorubicin. Nanoparticles targeted with CD38pep or CD138pep were prepared loaded with a doxorubicin pro-drug, and their in vivo efficacy was tested against that of free doxorubicin in a subcutaneous xenograft mouse model. Mice were injected with H929 cells and tumors allowed to grow to a predetermined size before IV injection of nanoparticle formulations began on day 1. Mice were injected with 3 mg/kg of doxorubicin or nanoparticle pro-drug equivalent on days 1, 3, 5, 7, and 9. Tumor volume (**a**), survival (**b**), and mouse weight (**c**) were tracked with mice being killed when tumor volume grew too large or mouse weight too low. After death or at the end of the study, organs were dissected and weighed (**d**). *n* = 6 for all groups and data represents means (± s.e.)

Efficacy of each treatment was determined by measuring tumor volume over the course of the study (Fig. 7a). Tumors belonging to mice in the PBS group grew unchecked, and all were killed by day 19 as a result of their tumors becoming too large. The least efficacious treatment was the CD138-targeted nanoparticles. This was not surprising due to the low tumor accumulation observed in the earlier biodistribution studies. The next lower efficacy treatment was the nontargeted nanoparticles. Interestingly, in previous studies the nontargeted nanoparticles showed higher accumulation than the CD138-targeted nanoparticles, but lower tumor cell uptake. This result raises a couple points, either that it is more important to deliver doxorubicin to the tumor site than to make sure it is inside the tumor cells or that the binding-site barrier greatly protects the inner part of the tumor, even if average tumor cell uptake is relatively high. Finally, the most efficacious treatment was with the CD38-targeted nanoparticles, suppressing tumor growth the most among all groups tested. The free doxorubicin group showed an efficacy between that of the CD38-targeted and nontargeted groups while the mice were alive, but all of the group had to be killed by day 11 in accordance with IACUC rules due to drastically declining health and body weight.

Interestingly, of all groups tested, only the group of mice injected with CD38-targeted nanoparticles had all members survive until the end of the study (Fig. 7b). Twenty-five percent of mice in the nontargeted group, 75% of the CD138-targeted group, and 100% of the PBS group were killed before the end of the study due to excessive tumor volume. Mouse weight was tracked to gauge overall health. All groups remained at approximately equivalent weights outside of the free doxorubicin group (Fig. 7c). Since all mice in the free doxorubicin group were killed by day 11 at the latest, while no other group showed any decrease in body weight, this demonstrated that the nanoparticle formulations were drastically less toxic than that of the free drug and accomplished the goal of delivering the drug payload to the tumor while avoiding harmful side effects. This outcome was supported by the dissection of the mice post-collection, wherein all the nanoparticle formulations displayed equal organ weights to that of the PBS control mice, while the mice dosed with free doxorubicin displayed significantly smaller kidneys, spleens, and livers (Fig. 7d). Of all the groups, the CD38-targeted nanoparticle-treated animals performed the best by a wide margin. They inhibited tumor size the most and displayed no systemic toxicity. These results are extremely promising for a drug delivery formulation that could prove to be a potent treatment in treating tumors where CD38 is overexpressed.

## Discussion

In this study, we synthesized and tested novel drug-loaded, CD38- and CD138-targeted nanoparticles for the treatment for multiple myeloma. We assessed binding, uptake, and cytotoxicity to multiple myeloma cells in vitro as well as biodistribution and efficacy in an in vivo mouse model. From these results, the most effective nanoparticle formulation was determined to be using CD38-targeting. This formulation, consisting of 50 nm liposomes with 0.7% CD38-targeting peptide density with an EG6 linker and an oligolysine sequence length of three, achieved 45% higher accumulation and fivefold higher tumor cell uptake when compared to nontargeted nanoparticles; twofold higher accumulation and tenfold higher uptake when compared to free doxorubicin; and the greatest efficacy in reducing tumor growth of all groups without showing any detectable signs of systemic toxicity. Certain particulars of the results demand detailed discussions and emphasis, including (1) that the CD38-targeted formulation was able to outperform both the free drug and the Doxil-similar nontargeted nanoparticles, (2) that the CD38-targeted formulation performed much better compared to the CD138-targeted version in vivo, although targeting CD138 resulted in much higher binding and uptake in vitro, and (3) that the best performing CD38-targeted formulation turned out not to be the one with the highest load of targeting peptide.

The most important result was that CD38-targeted nanoparticles outperformed both free doxorubicin and nontargeted nanoparticles. Outperforming doxorubicin, which is one of the World Health Organization’s Essential Medicines, is a noteworthy accomplishment. The CD38-targeted nanoparticles displayed a much improved biodistribution profile than free doxorubicin by improving drug accumulation at the tumor site while reducing its accumulation at major organs. This resulted in drastically lowered systemic toxicity, which is supported by almost no change in body weight across all the nanoparticle groups, while in the free doxorubicin group, all the mice had to be killed by day 11 due to weight loss. The biodistribution data also showed that the nontargeted nanoparticles increased drug accumulation at the tumor site by approximately 60%, which is in agreement with previous studies [33, 34]. Since the CD38-targeted formulation improved efficacy over the nontargeted nanoparticles, these results demonstrate their potential as a new treatment option over the current standard treatment Doxil in multiple myeloma cases that overexpress CD38.

Another significant result was that although the CD138-targeted nanoparticles had much higher binding and uptake in vitro, they performed much worse in vivo than not only the CD38-targeted particles but even the nontargeted nanoparticles. The excessively poor accumulation of the CD138-targeted nanoparticles in the tumors was perhaps related to the nonselective binding to healthy cells that also express CD138 receptors. We hypothesize that CD138-targeted nanoparticles bound particularly well to healthy circulating lymphocytes, causing their depletion and reducing fraction of particles that reached their intended destination of the cancer cells. As the treatment is delivered via IV, the targeted nanoparticles will encounter a large number of healthy cells *en route* to the tumor site. Due to the CD138-targeted nanoparticles having significantly more rapid binding and uptake, these particles are more prone to off-target delivery than the CD38-targeted nanoparticles. Another factor that may affect this off-target uptake is the activity of the receptor itself. If the CD138 receptor actively triggers endocytosis, then the CD138 nanoparticles could trigger rapid cellular uptake at a higher rate than CD38, leading to less selectivity for the tumor cells and more off-target losses. If this is the case, this roadblock cannot easily be engineered around by simple design alterations such as reducing monovalent affinity of targeting peptide or avidity of the nanoparticle, as it is an inherent property of the targeted receptor. Since a similar drop in tumor accumulation at higher peptide densities due to off-target effects has been observed with other targeting peptides such as VLA-4, this type of reduced tumor accumulation is not unprecedented [16].

Finally, also noteworthy was that the optimal CD38pep density was not at the highest peptide loading. Although all CD38-targeted nanoparticles showed similar accumulation at the tumor site, tumor cell uptake increased with peptide density up to 0.5% CD38 and then sharply decreased at 1%. This result was likely due to the binding-site barrier phenomenon, wherein a large fraction of the targeted molecules or particles will bind to just the first layer of cells directly surrounding the capillaries within a tumor, with almost none of it making it deeper within the tissue. We then tested a narrower range of peptide densities between these two values in vitro to see whether we could observe anything of note. In this test, we found a sharp increase in uptake between 0.7% and 0.8% CD38pep loading. We speculate that this spike is likely due to the physical relationship of the average distance between the peptides on the nanoparticles and the receptors on the targeted cells [50, 51]. Since in vivo tumor cell uptake decreased when CD38pep density increased from 0.5 to 1%, we formulated nanoparticles that had 0.7% peptide density in order to remain below the critical point at 1% which corresponded to a significant decrease in vivo uptake.

## Conclusions

The results from this study established that CD38-targeted nanoparticles have strong potential for clinical treatment for multiple myeloma. Compared to upcoming CD38 antibody treatments, described formulation potentially offers a targeted and more cell-selective approach of drug delivery by utilizing multivalent low-affinity interactions to reduce off-target binding to cells displaying healthy amounts of CD38. In turn, lowered off-target uptake would result in fewer adverse effects and allow use of increased dosing to improve elimination of cancer cells to deliver higher efficacy while using the same drug [17, 32]. This treatment strategy may possibly be relevant to other types of cancer as well. Cancers that overexpress a specific receptor that simultaneously exists on healthy cells make an ideal target for similar strategies, such as CD20 for B-cell lymphoma or CD30 for Hodgkin’s lymphoma. Our laboratory has also recently developed nanoparticles for selective uptake of CD22 overexpressing B-cell malignancies and HER2 overexpressing breast cancer, proving both to be other possible targets for liposomes designed through the same platform [52, 53]. Further preclinical evaluation is needed, such as dosing studies on how much can be given without causing systemic toxicity, and if tumor remission can be achieved with a significantly high dose. Additional nanoparticle elements, such as dual-targeting with a second targeting peptide, may also increase selectivity and could reduce off-target effects at high doses. Another option is the addition of an endosomal escape element such as a cell-penetrating peptide or via the proton sponge effect to ensure that more of the drug is released from the endosome to the rest of the cell. Even without these elements, however, the CD38-targeted nanoparticles used in this study delivered enhanced efficacy and safety over current treatment options and hold the potential to greatly improve patient outcome.

## Supplementary information

**Additional file1**. Peptide density fine tuning for uptake of CD38pep targeted nanoparticles.

## Data Availability

The datasets used and analyzed during the current study are available from the corresponding author on reasonable request.

## Abbreviations

ACN: Acetonitrile
BSA: Bovine serum albumin
CAM-DR: Cell adhesion-mediated drug resistance
DCM: Dichloromethane
DiD: 1,1′-Dioctadecyl-3,3,3′,3′-tetramethylindodicarbocyanine perchlorate
DIEA: *N,N*-Diisopropylethylamine
DLS: Dynamic light scattering
DMF: Dimethylformamide
Dox: Doxorubicin
DSPC: 1,2-Distearoyl-*sn*-glycero-3-phosphocholine
EG: Ethylene glycol
EPR: Enhanced permeability and retention
FBS: Fetal bovine serum
FDA: Food and drug administration
FITC: Fluorescein isothiocyanate
HBTU: 2-(1H-benzotriazol-1-yl)-1,1,3,3-tetramethyluronium hexafluorophosphate
IPA: Isopropanol
MALDI: Matrix-assisted laser desorption/ionization
MM: Multiple myeloma
MS: Mass spectroscopy
NOD–SCID: Nonobese diabetic/severe combined immunodeficiency
PBS: Phosphate buffered saline
PEG: Polyethylene glycol
RP-HPLC: Reversed-phase high-performance liquid chromatography
SCID: Severe combined immunodeficiency
TFA: Trifluoroacetic acid
TIS: Triisopropylsilane
TOF: Time-of-flight
