# Supplementary material for: In vivo evaluation of CD38 and CD138 as targets for nanoparticle-based drug delivery in multiple myeloma
Source: J Hematol Oncol. 2020 Nov 2;13:145. doi: 10.1186/s13045-020-00965-4 (PMC7607744; doi:10.1186/s13045-020-00965-4)
Supplement: Supplementary file 1 — Additional file1. Peptide density fine tuning for uptake of CD38pep targeted nanoparticles. [file 13045_2020_965_MOESM1_ESM.docx]

Additional File 1

*In vivo* Evaluation of CD38 and CD138 as Targets for Nanoparticle-Based

Drug Delivery in Multiple Myeloma

David T. Omstead, Franklin Mejia, Jenna Sjoerdsma, Baksun Kim, Jaeho Shin, Sabrina Khan, Junmin Wu, Tanyel Kiziltepe, Laurie E. Littlepage, and Basar Bilgicer^*^


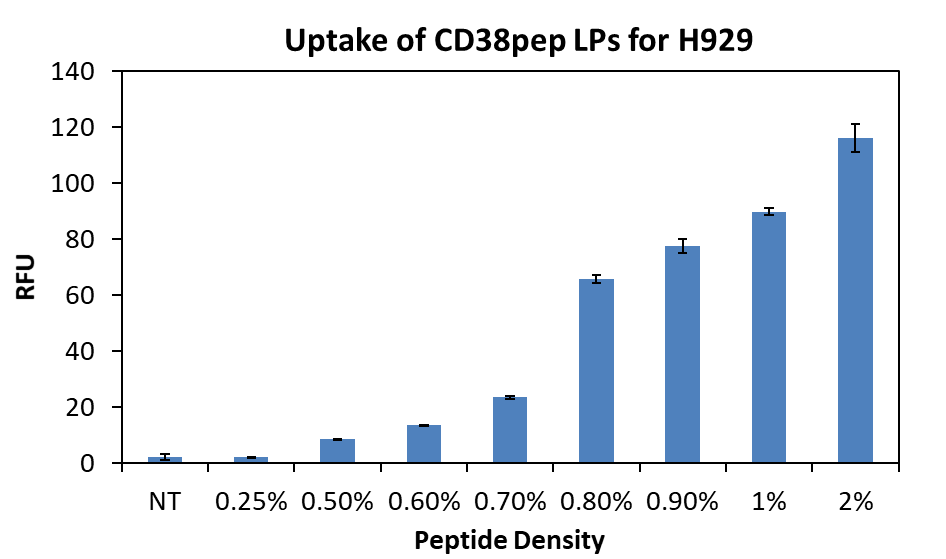


**Figure S1.** Peptide density fine tuning for uptake of CD38pep targeted nanoparticles. Nanoparticles were incubated with H929 cells in media for 3 hours, trypsinized to remove nanoparticles bound to the surface but which had not yet undergone cellular uptake, and then fluorescence was measured by flow cytometry. All experiments were done in triplicate. Data represents means (±s.d.).
